# Characterization of the 5-HT_2C_ receptor agonist lorcaserin on efficacy and safety measures in a rat model of diet-induced obesity

**DOI:** 10.1002/prp2.84

**Published:** 2014-11-07

**Authors:** Guy A Higgins, Jill Desnoyer, Annalise Van Niekerk, Leo B Silenieks, Winnie Lau, Sandy Thevarkunnel, Julia Izhakova, Ines AM DeLannoy, Paul J Fletcher, Josepha DeLay, Howard Dobson

**Affiliations:** 1 InterVivo Solutions Inc.120 Carlton St., Toronto, Ontario, Canada, M5A 4K2; 2 Department of Pharmacology & Toxicology, University of TorontoToronto, Ontario, Canada; 3 Section of Biopsychology, Clarke Division, Centre for Addiction and Mental Health250 College St., Toronto, Ontario, Canada, M5T 1R8; 4 Department of Psychiatry & Psychology, University of TorontoToronto, Ontario, Canada; 5 Animal Health Laboratory, U. GuelphGuelph, Ontario, Canada; 6 CanCog Technologies Inc.120 Carlton St., Toronto, Ontario, Canada, M5A 4K2; 7 Department of Clinical Studies, University of GuelphGuelph, Ontario, Canada; 8 Department of Biomedical Physics, University of Western OntarioLondon, Ontario, Canada

**Keywords:** Biomarkers, echocardiography, efficacy, lorcaserin, obesity, safety, valvulopathy

## Abstract

The 5-HT_2C_ receptor agonist lorcaserin (Belviq®) has been Food and Drug Administration (FDA) approved for the treatment of obesity. The present study is a back translational investigation into the effect of 28-day lorcaserin treatment in a diet-induced obesity (DIO) model using male, Sprague–Dawley rats. An assessment of drug effect on efficacy and multiple safety endpoints including cardiac function was undertaken. Lorcaserin (1–2 mg/kg SC b.i.d.) significantly reduced percentage body weight gain compared to vehicle-treated controls (VEH: 10.6 ± 0.4%; LOR 1: 7.6 ± 1.2%; LOR 2: 5.4 ± 0.6%). Measurement of body composition using quantitative magnetic resonance (QMR) imaging indicated this change was due to the selective reduction in body fat mass. Modest effects on food intake were recorded. At the completion of the treatment phase, echocardiography revealed no evidence for valvulopathy, that is, no aortic or mitral valve regurgitation. The pharmacokinetics of the present treatment regimen was determined over a 7-day treatment period; plasma *C*_min_ and *C*_max_ were in the range 13–160 ng/mL (1 mg/kg b.i.d.) and 34–264 ng/mL (2 mg/kg b.i.d.) with no evidence for drug accumulation. In sum, these studies show an effect of lorcaserin in the DIO model, that in the context of the primary endpoint measure of % body weight change was similar to that reported clinically (i.e., 3.0–5.2% vs. 3.2%). The present studies highlight the translational value of obesity models such as DIO, and suggest that assuming consideration is paid to nonspecific drug effects such as malaise, the DIO model has reasonable forward translational value to help predict clinical outcomes of a new chemical entity.

## Introduction

Obesity is the leading cause of preventable death worldwide, being a causal factor in multiple serious clinical conditions, including type 2 diabetes, dyslipidemia, atherosclerosis, hypertension, stroke, certain cancers, as well as reduced affect, sleep apnea, and osteoarthritis (Heal et al. 2009, 2013; Powell et al. 2011). The medical need to treat obesity is significant, in the US alone approximately 35% adults are currently classified as obese with very limited pharmaco-therapeutic options (Flegel et al. 2010; Powell et al. 2011). When considering the approval of a new drug to treat obesity, regulatory agencies such as the Food and Drug Administration (FDA) and EMA, require not only significant weight loss (typically 5% from a placebo-corrected baseline), but also improvement to comorbidities such as lipid content, glycemia, and blood pressure (Heal et al. 2009; Kennett and Clifton 2010).

For several years until 1997, the 5-hydroxytryptamine (serotonin) (5-HT) releaser/reuptake inhibitor (dex)fenfluramine was used successfully for the treatment of obesity. Unfortunately, (dex)fenfluramine was withdrawn due to the cardiovascular complications, including valvulopathy and pulmonary hypertension (Connolly et al. 1997). The clinical efficacy of (dex)fenfluramine, prompted efforts to establish the specific receptor target(s) which mediated the anorectic effect, with the prediction that this could be distinguished from the mechanisms underlying the cardiovascular effects. This prediction was realized with the discovery of a primary role for the 5-HT_2C_ receptor in the effects on feeding and body weight (Vickers et al. 1999, 2001; Heisler et al. 2002), while 5-HT_2B_ receptor activation likely contributes to the cardiovascular safety issues (Hutcheson et al. 2011 for review). Together with parallel investigations into how 5-HT systems affected feeding processes (Blundell and Halford 1998), this prompted a search for functionally selective 5-HT_2C_ receptor agonists as novel antiobesity agents.

In 2012, the FDA approved lorcaserin as the first selective 5-HT_2C_ receptor agonist drug for the treatment of obesity. Approval was based on the positive outcomes from two large pivotal Phase III trials conducted in a “normal” obese population (Smith et al. 2010; Fidler et al. 2011), and a third trial conducted in a cohort with type 2 diabetes (O'Neil et al. 2012). A meta-analysis of these data (Chan et al. 2013) demonstrated a 3.32 kg weight loss compared to placebo, which was almost equivalent to (dex)fenfluramine (Kennett and Clifton 2010; Heal et al. 2013). Modest benefits on secondary outcome measures including blood pressure and total cholesterol, LDL-C, and triglycerides were also found (Chan et al. 2013). Importantly, on the evidence gained from clinical trials conducted to date in approximately 3500 adults treated with the approved 10 mg b.i.d regimen for at least 1 year, lorcaserin does not appear to induce the cardiac valvulopathy or pulmonary hypertension associated with use of (dex)fenfluramine (Weissman et al. 2013).

Animal models of obesity are important for the identification of new treatments. However, despite the publication of multiple clinical studies (Smith et al. 2009, 2010; Fidler et al. 2011; O'Neil et al. 2012), there is relatively little preclinical data on the effects of lorcaserin in obesity models. Two reports have described the effect of 28-day lorcaserin treatment in Sprague–Dawley rats fed regular diet (Smith et al. 2008), and in Levin rats fed high-fat diet (Thomsen et al. 2008). While both studies revealed lorcaserin reduced food intake and body mass, the doses producing those effects (9–36 mg/kg b.i.d, Smith et al. 2008; 4.5–18 mg/kg b.i.d, Thomsen et al. 2008) were high compared to other reports (0.3–3 mg/kg; Levin et al. 2011; Higgins et al. 2012, 2013a) and at a dose-range where effects such as motor changes and malaise may have contributed to apparent efficacy. It was the purpose of the present study to evaluate lorcaserin at doses more consistent with these other studies (i.e., 1–2 mg/kg SC b.i.d) on food intake and body mass in a diet-induced obesity (DIO) model. Furthermore, the effect of lorcaserin on plasma lipid profile, fat versus lean mass and cardiac function using echocardiogram measures were investigated in this model. Also drug plasma levels attained by the treatment regimen over a 24 h period were measured. The primary intention of these studies was to enable a back translation to the earlier clinical studies reported for lorcaserin, and to compare with previous data we have obtained with this drug in other (acute) models of feeding behavior (Higgins et al. 2012, 2013a). Backtranslating clinical findings to the equivalent animal model is important for the purpose of validation and to determine the models value as a guide to efficient transition of an NCE from preclinical to clinical proof-of-concept testing (Enna and Williams 2009; Day et al. 2011).

## Materials and Methods

### Animals and housing

Male, Sprague–Dawley rats (Charles River, St. Constant, Quebec) were individually housed in solid bottom perspex cages (dimension: 19″ *L* × 10″ *D* × 8″ *H*) throughout the study course. Rats were approximately 7 weeks of age (body weight range: 170–200 g) at the start of the study. The animals were housed in a temperature and humidity controlled environment under a 12 h light:dark cycle (lights on 05:00–17:00 h). Twenty-six rats were fed a high-fat diet (Research Diets D12492; 5.24 kcal/g) throughout the study, while a further 10 rats were fed regular diet (LabDiet 5001: 4.07 kcal/g) over this period. All animal studies complied with the appropriate Institutional and Canadian Council on Animal Care (CCAC) guidelines relating to animal experimentation.

### Measurement of food/water intake, blood lipid content, and oral glucose challenge

Food and water intakes were measured by weighing the quantity of food in the hopper, or the water bottle each day – the difference score between days was assumed to be the quantity consumed. These intakes and body weight were measured at 14:00 ± 1:00 h.

Blood was collected by saphenous vein bleeds into lithium heparin tubes (Sarstedt Microvette CB300; Sarstedt Canada Inc., Saint Léonard, QC, Canada). Cholesterol and triglyceride content was measured by Antech Diagnostics (Mississauga, ON, Canada). Blood glucose was measured in-house using Accu-Check Aviva test strips and a glucometer (Accu-Chek Aviva, model type 0353231003; Roche Diagnostics, Mississauga, ON, Canada).

Animals were also periodically subjected to an oral glucose challenge. Following an overnight fast, blood was collected at 30 min pre, and immediately prior to, an oral glucose load (2 g/kg; 5 mL/kg dose volume). Blood was collected at 15, 30, 60, 120, 180, 240 min and blood glucose measured as previously described.

### Structural magnetic resonance imaging

**Figure 1:**
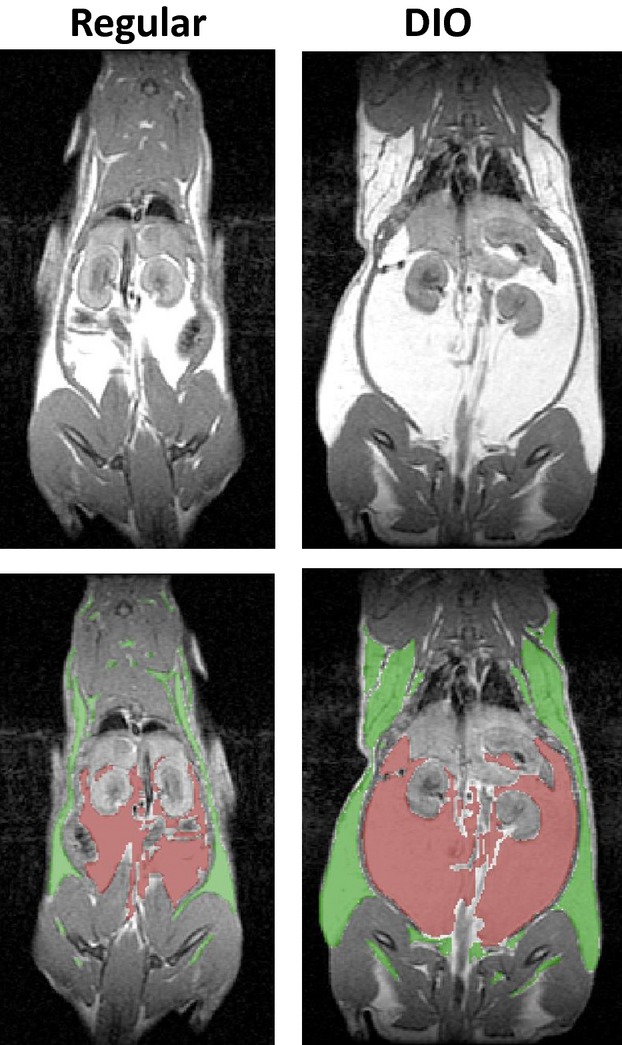
Whole body magnetic resonance imaging of a rat fed regular diet for 12 weeks and a rat fed high-fat diet (DIO) for the equivalent period. The lower figures show the segmentation of these images between the subcutaneous (green) and visceral (red) fat compartments.

Under light general anesthesia (Buprenorphine: 0.05–0.1 mg/kg, Midazolam: 1–2 mg/kg, and Dexmedetomidine: 0.02–0.05 mg/kg by the intramuscular route) whole body magnetic resonance images were collected using a 1.5 T magnet (GE Excite MRI system, Milwaukee, WI) and a human quadrature knee coil. Two separate series of images were collected; fat and water images with the central frequency centered on water and fat only images with the central frequency centered on fat. The image acquisition parameters were TR 1500 msec, TE 10 msec, and a 15 × 15 cm field of view with a 256 × 196 matrix in order to quantify visceral and subcutaneous fat volumes. The images were manually segmented into visceral and nonvisceral regions. An additional region of interest was manually drawn to include the liver. Ratio images and fat volumes were calculated as described by Johnson et al. (2008) in order to segment fat from nonfat tissues and using volume averaging correction total fat volume for each compartment was determined. Sample segmented images are presented in Figure1. Additionally, proton magnetic resonance spectroscopy (MRS) data were acquired from a 1 cm × 1 cm × 1 cm voxel in the liver using a standard PRESS sequence.

### Quantitative magnetic resonance imaging

Subjects were restrained in a Plexiglas tube which was inserted into the magnet bore (Echo MRI-B; Echo Medical Systems, Houston, TX). Data were collected over a 110-sec scan time, and the animals remained fully conscious throughout this period. The average of two such readings were taken for each rat. The maximal size of rat that could be accommodated within the restraining tube, the diameter of which was constrained by the magnet bore was approximately 1 kg. Quantitative magnetic resonance (QMR) uses similar technology to conventional MRI, but rather than an image it provides quantitative body composition data. The QMR technique has been validated in rats against chemical carcass composition (Johnson et al. 2009). It provides estimates of fat and lean mass and free and total body water mass, but unlike Dual energy x-ray absorbtiometry (DEXA), it does not provide data regarding bone mass.

### Echocardiography

General anesthesia was induced and maintained with gaseous isoflurane in oxygen. The left chest wall was close clipped and coupling medium (Aquasonic Ultrasound Gel; CDMV, Montreal, QC) applied. An ultrasound machine (MyLab Alpha, Esaote Canada, Georgetown, ON) with a 7/3 MHz phased array transducer was used for echocardiographic evaluation. A left parasternal approach was used and long- and short-axis views of the heart were captured. Color flow and pulsed Doppler interrogation of the aortic and mitral valves was performed. Images were reviewed off line for evidence of aortic and mitral valve regurgitation.

### Initial characterization of the DIO model

The initial part of this study involved a characterization of the DIO model and the primary endpoints that were integral to the treatment phase of the study. A total of 26 rats were fed a high-fat diet (Research Diets D12492) for 3 months, while a further 10 rats were fed regular diet (LabDiet 5001) for the equivalent period. The 10 ad-lib regular diet rats, served as controls to characterize the DIO model, otherwise data from these animals are not reported in the current study. Body weights and food/water intakes were measured weekly. At 6 and 12 weeks, additional measures of body composition (QMR), and blood lipid content was measured, and an oral glucose tolerance test (OGTT) was conducted. Because QMR only provided a measure of total fat content, MRI of a single representative rat from each of the two diet groups was conducted at 12 weeks to examine the distribution of fat between the visceral and subcutaneous compartments and to measure fat content of the liver.

### Treatment phase

Following characterization of the DIO rats, all 26 rats fed the high-fat diet for 3 months were allocated into 3 groups balanced for equivalent body weight, food/water intake, and fat mass. Designated groups were vehicle SC b.i.d (*n* = 10 rats), lorcaserin 1 mg/kg SC b.i.d (*n* = 8), and lorcaserin 2 mg/kg SC b.i.d (*n* = 8). Lorcaserin hydrochloride (Hangzhou Trylead Chemical Technology Co., Hangzhou, China) was dissolved in saline and administered in a dose volume of 1 ml/kg, Lorcaserin dose was expressed as that of the base.

Initially over a 3-day period, all rats received twice daily SC saline injections for familiarization purposes. Next the 28-day treatment phase began. During this period all rats were allowed ad-lib access to high-fat diet and water. Body weight and food/water intakes were measured daily at approximately 14:00 h. Treatments were administered at approximately 08:00 h and 17:00 h, that is, just prior to dark phase onset. At the completion of the 28-day treatment phase the animals were reassessed for body composition through QMR, and blood was collected for measurement of lipid content, clinical chemistry, and glucose response to an oral glucose load. These studies were completed over 5 days immediately following cessation of drug treatment. At 7 and 14 days post cessation of drug treatment both food/water intake and body weight was recorded to determine these measures at washout.

### Safety phase

Blood was collected for clinical chemistry and cell counts. On day 21 and 22 post cessation of drug treatment, all rats underwent echocardiography. At the completion of the 22 day washout phase, the rats were sacrificed and organ weights were checked for gross appearance and weighed. Cardiac tissue was also taken for histology purposes. The tissue was flushed with saline and suspended in a solution of 10% neutral-buffered formalin. Formalin-fixed hearts were sectioned longitudinally in a plane from the base of the aorta through the apex, and the ventral hemisections were processed and paraffin embedded for light microscopy. Three 4 *μ*m sections taken at 100 *μ*m intervals were stained with hematoxylin and eosin. Additional sections at 100 *μ*m intervals were examined in those cases where original sections did not capture aortic and right and left atrioventricular valves. Histologic examination was carried out by a single board certified pathologist (JDeL), and valves were scored individually on the basis of location and composition of valvular thickenings or other abnormalities.

In a parallel study, a cohort of Sprague–Dawley rats were treated once daily with 5-HT creatinine sulfate (4 days 75 mg/kg i.p. × 1 daily, followed by 24 days 60 mg/kg i.p.  × 1 daily) or saline control. This treatment schedule was adopted based on the experimental evidence that it would elicit a cardiac valvulopathy (Gustafsson et al. 2005; Elangbam et al. 2008), that is, a control for the lorcaserin study. At the completion of this treatment phase, all rats underwent echocardiography, followed by sacrifice and removal of cardiac tissue for histology. These rats were maintained on regular diet (i.e., LabDiet 5001) and water for the study duration.

### Pharmacokinetic study

Eight rats which had previously received lorcaserin during the 28-day treatment phase were allowed to remain on the high-fat diet for 8 weeks to allow adequate washout of drug. After this time, the rats were randomly allocated into 2 groups of 4, one group designated lorcaserin 1 mg/kg SC b.i.d and the other lorcaserin 2 mg/kg SC b.i.d. Rats were dosed to a schedule similar to that adopted for the feeding study, that is, 2× daily, 10 h interval between the daytime doses. On day 1 and day 7, blood was collected from the saphenous vein at timepoints pre-dose, 0.25 h, 0.5 h, 1 h, 4 h, 8 h after each daily dose. This allowed comparison between drug exposure on day 1 and day 7 and provided an estimate of drug exposure over the treatment phase of the study. All rats remained on the high-fat diet for the duration of this study phase.

Bioanalytical analyses were conducted using an AB SCIEX API4000 liquid chromatography-mass spectrometry/mass spectrometry (LC-MS/MS) system (AB SCIEX, Framingham, MA). Lorcaserin was measured based on the methods a**s** previously described (Higgins et al. 2013a).

### Statistical analysis

All statistical analyses for experiments were completed using Statistica Version 11 (StatSoft, Tulsa, OK). Analysis of variance (ANOVA) with repeated measures was utilized with treatment as the grouping factor and time as the repeated measure for food and water intake, body weight, and body composition and specific biochemistry measures (glucose, cholesterol, and triglycerides). Single factor ANOVA were completed for the OGTT area under curves (AUCs) determined by the trapezoidal method, QMR measurements, organ weights, and the 28-day safety study with treatment as the grouping factor. Tukey's HSD was used as the post-hoc analysis when further group comparisons were warranted. Statistical significance was set at *P* < 0.05, and at *P* < 0.1 a trend toward significance was considered.

## Results

### Characterization of the DIO model

A summary of the principal findings from this study phase is shown in Table1. Prior to placement on the study diets, the DIO and regular diet groups did not differ in body weight (*t*(34) = 1.0, NS) all animals being in the range 170–200 g. At this body weight and age, the percentage lean and fat mass was in the range 80.9 ± 0.4% (lean mass) and 7.6 ± 0.3% (fat mass).

**Table 1:** Comparison between rats fed a regular diet (Lab Diet 5001) and a high-fat diet (Research diet D12492) on measures of food and water intake (determined by mean over 3 days), body composition as measured by QMR, blood glucose, cholesterol (Chol) and triglyceride (TG) content, and response to an oral glucose load measured as AUC (OGTT)

		Body weight (g)	Food intake (g)	Water intake (g)	Body composition				Blood glucose (mmol/L)		OGTT (mmol/L × min)	Chol (mmol/L)	TG (mmol/L)
					Fat (g)	Lean (g)	Fat (%)	Lean (%)	Unfasted	Fasted			
6 weeks	Regular	496 ± 11	38.0 ± 1.0	58.0 ± 2.8	48.2 ± 2.6	381.6 ± 8.1	9.7 ± 0.4	76.9 ± 0.3	6.5 ± 0.3	5.9 ± 0.3	1733 ± 43	2.2 ± 0.1	2.2 ± 0.2
	DIO	557 ± 11*	23.3 ± 0.6*	39.0 ± 2.2*	93.1 ± 5.9*	385.6 ± 7.0	16.7 ± 0.9*	69.4 ± 1.2*	6.8 ± 0.2	5.8 ± 0.3	1957 ± 45*	2.9 ± 0.1*	2.0 ± 0.2
12 weeks	Regular	595 ± 16	32.1 ± 1.1	56.3 ± 2.4	68.2 ± 4.2	438.4 ± 11.0	11.4 ± 0.6	73.7 ± 0.6	6.2 ± 0.1	6.6 ± 0.3	1940 ± 32	2.4 ± 0.1	2.4 ± 0.3
	DIO	702 ± 12*	20.0 ± 0.5*	36.0 ± 1.5*	131.3 ± 5.0*	434.4 ± 5.9	18.7 ± 0.6*	62.2 ± 1.0*	7.0 ± 0.1*	6.3 ± 0.2	2194 ± 79*	3.2 ± 0.1*	2.0 ± 0.2

* *P* < 0.05 versus regular diet controls at equivalent timepoint.

After 6 weeks on the study diets, the DIO group had a significantly greater body weight, fat content (measured either as % fat or g fat), blood cholesterol, and an elevated response to an oral glucose load compared to rats fed regular diet (see Table1). Since lean mass was not increased, the gain in body weight was essentially due to increased adiposity. Actual food and water consumption was lower compared to rats fed a regular diet. At this stage, there were no group differences in blood glucose (fasted and unfasted) or triglyceride levels.

After 12 weeks, the DIO group showed similar changes to that seen at 6 weeks although with slightly increased magnitude and significance. The only qualitative difference from 6 weeks, was a significant elevation in unfasted blood glucose levels (see Table1). Some variability was evident across the rats fed the high-fat diet, consistent with individual rats showing differences in susceptibility to obesity (Ghibaudi et al. 2002). For example fat content ranged from 11.6% to 24.0% across all DIO rats, compared to 8.4–13.5% in rats fed regular diet. Whole body MRI images were taken of a representative DIO rat and a regular diet control rat after 12 weeks on diet to determine the fat content between the visceral and subcutaneous compartments (see Fig.1). Measurement of fat volumes from the visceral compartment were 38,104 mm^3^ versus 180,470 mm^3^, and from the subcutaneous compartment were 116,430 mm^3^ versus 254,490 mm^3^ (regular vs. DIO). This translated to a difference in relative fat distributions with the DIO rat having 41.5% fat in the visceral compartment compared to 24.7% in the rat fed regular diet. The MRS data demonstrated a liver fat content of 19.7% and 3.8% between the DIO and regular diet groups respectively.

### Effect of 28-day lorcaserin treatment in the DIO model – efficacy

**Figure 2:**
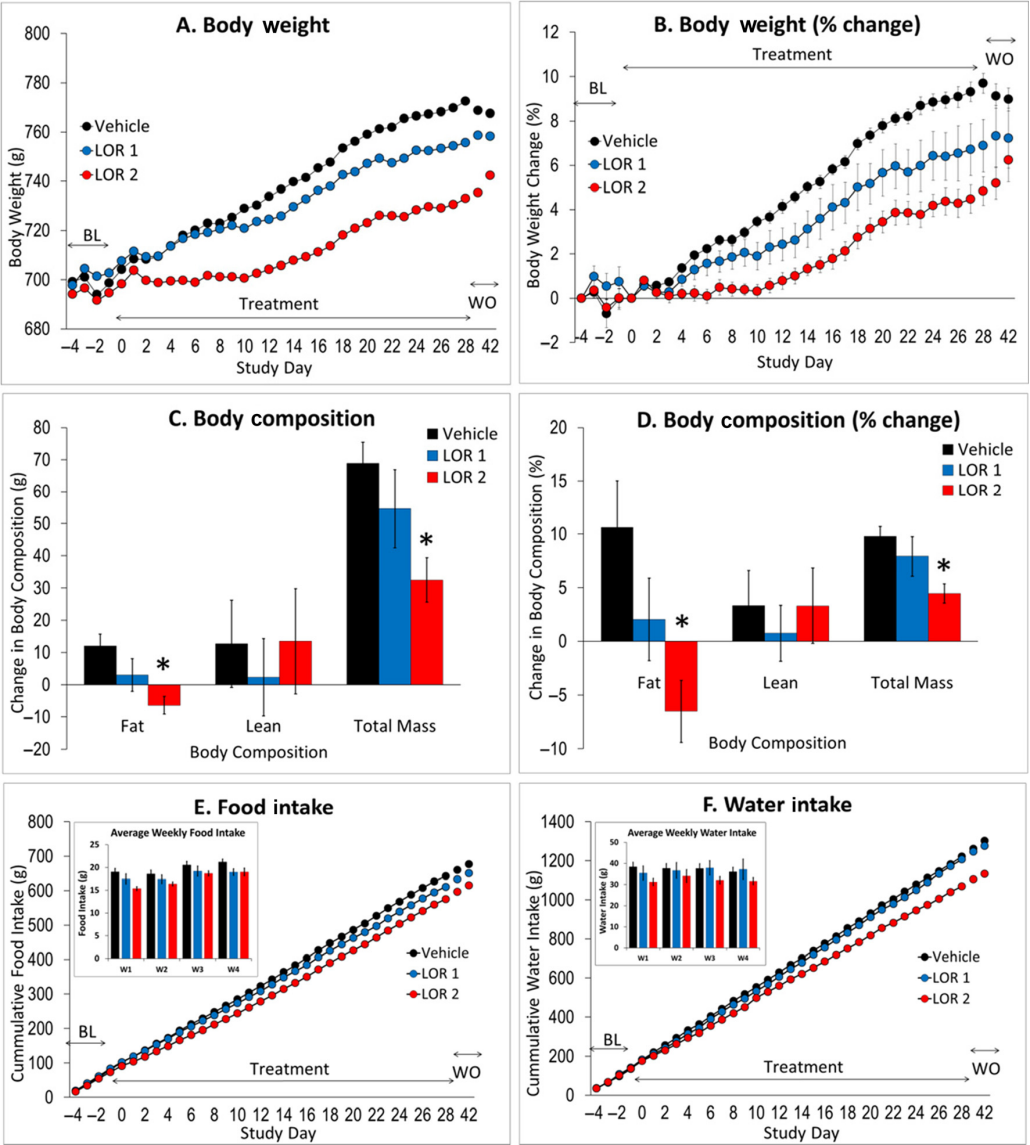
(A) Effect of vehicle or lorcaserin (1–2 mg/kg SC b.i.d.) on body weight measured over 3 days baseline (BL), 28-day treatment, and 2 weeks washout phase (WO). (B) represents the conversion of body weight, to percent change compared to pretreatment baseline. This conversion shows that at 2 mg/kg b.i.d., lorcaserin reduced % body weight by approximately 5.2% compared to vehicle controls. (C) Measurement of body composition, that is, fat or lean mass, was made using QMR and a difference value was determined between a reading made 6 days before treatment phase, and 1 day after the treatment phase. (D) represents the conversion of body composition, to percent change compared to pretreatment baseline. (E) Cumulative food, and (F) water intake, measured over the baseline, 28-day treatment and, washout phase. In figure (E) and (F) the inset shows the daily intakes averaged for each animal by week. *N* = 8–10 rats per group, all rats fed high-fat diet (Research Diet D12492; 5.24 kcal/g). *P < 0.05 versus vehicle.

At baseline, over the 4 pretreatment days prior to the onset of drug treatment, all 3 groups fed the high-fat diet had equivalent body weights (*F*_2,23_ = 0.03, NS), daily food, and water intakes (*F*_2,21_ ≤ 0.4, NS) (see “baseline” on Fig.2A, B, E, and F).

Over the 28 days of treatment, vehicle-treated rats gained approximately 70 g (10.6 ± 0.4%) of body weight (see Fig.2A and B). Lorcaserin (1–2 mg/kg b.i.d)-treated rats showed a dose-related reduction in body weight gain relative to vehicle-treated controls, such that percentage weight gain in the 1 mg/kg b.i.d group was 7.6 ± 1.2%, and in the 2 mg/kg b.i.d group was 5.4 ± 0.6%. Overall analysis of the 28 days of treatment revealed a significant treatment × days interaction both on body weight (*F*_54,644_ = 6.2, *P* < 0.01) and percentage body weight change (*F*_54,644_ = 6.4, *P* < 0.01). By treatment day 12, the percentage body weight change was significantly different between VEH and 2 mg/kg LOR groups and remained so for the duration of this treatment. On no days were any significant group differences evident between Veh and 1 mg/kg LOR groups (see Fig.2A and B).

Daily food intake was reduced by lorcaserin treatment, although the magnitude of this effect was small and essentially confined to the 2 mg/kg dose (see Fig.2E). The effect of lorcaserin on cumulative food intake was affected in a time-dependent manner over the 28-day treatment period (treatment: *F*_2,23_ = 4.1; *P* < 0.05; treatment × day: *F*_56,644_ = 2.7; *P* < 0.01). To examine the time dependency, a separate analysis was conducted to examine average intake by week (Fig.2E inset). A main effect of week (*F*_3,69_ = 17.7, *P* < 0.01) was found, although treatment narrowly missed significance (*F*_2,23_ = 2.8; *P* = 0.08). The clearest effect of lorcaserin on food intake was evident during the first week, and confining the analysis to week 1 revealed a main effect of lorcaserin (*F*_2,23_ = 5.4, *P* = 0.01) (see Fig.2E inset). Analysis of weeks 2, 3, or 4 failed to show a main effect of lorcaserin (*F*_2,23_ < 1.9, NS). Equivalent analyses on water intake identified no main effect of treatment (*F*_2,23_ = 1.8, NS) although a significant treatment x day interaction was found (*F*_56,644_ = 1.6, *P* < 0.01). Again this seemed to reflect a predominant effect of lorcaserin treatment to reduce intake during week 1 (see Fig.2F).

On body composition, lorcaserin produced a significant decrease in fat mass measured either in grams (*F*_2,23_ = 5.6; *P* = 0.01), or as percent change (*F*_2,23_ = 5.0; *P* < 0.05) (see Fig.2C and D). In each case only the 2 mg/kg LOR group was significantly different to vehicle. In contrast there was no effect of lorcaserin on lean mass (grams: *F*_2,23_ = 0.2, NS; % change: *F*_2,23_ = 0.2, NS). Total mass was also reduced (grams: *F*_2,23_ = 4.5, *P* < 0.05; % change: *F*_2,23_ = 4.7, *P* = 0.01) (see Fig.2C and D).

Lorcaserin modestly affected plasma lipid levels (see Table2). Although there was no main effect of treatment on either blood cholesterol, triglyceride, or glucose (fasted) levels including pre- versus posttreatment as a factor (treatment: *F*_2,23_ < 0.5, NS; treatment × time: *F*_2,23_ < 1.8, NS), restricting comparisons to day 28 identified a significant lowering of cholesterol by lorcaserin compared to vehicle pretreatment (see Table2). All groups had equivalent cholesterol levels at baseline.

**Table 2:** Effect of 28-day lorcaserin treatment on blood glucose and lipid biomarkers in the DIO rats

		Blood glucose (fasted) (mmol/L)	OGTT (mmol/L × min)	Cholesterol (mmol/L)	Triglycerides (mmol/L)
Vehicle	Pre	6.19 ± 0.30	–	3.35 ± 0.16	1.95 ± 0.20
	28 day	6.11 ± 0.24	2036 ± 56	3.13 ± 0.09	2.29 ± 0.34
Lorcaserin 1 mg/kg	Pre	6.46 ± 0.25	–	3.17 ± 0.19	1.91 ± 0.21
	28 day	6.16 ± 0.19	2133 ± 117	2.74 ± 0.11*	1.68 ± 0.12
Lorcaserin 2 mg/kg	Pre	6.40 ± 0.42	–	3.06 ± 0.26	2.13 ± 0.40
	28 day	5.85 ± 0.17	1992 ± 62	2.71 ± 0.11*	1.80 ± 0.30

OGTT, oral glucose tolerance test.

* *P* < 0.05 versus 28 vehicle-treated group (28 day).

### Effect of 28-day lorcaserin treatment in the DIO model – safety

At the completion of 28-day treatment with lorcaserin at 1 and 2 mg/kg, blood was collected for analysis of total blood cell counts and clinical chemistry markers. The clinical chemistry results are summarized in Table3. There were no significant effects of drug treatment on any marker which all tended to fall within the normal range for Sprague–Dawley rats (Petterino and Argentino-Storino 2006). Blood counts were also unaffected by treatment (RBC (10^12^/L): Veh: 8.0 ± 0.3, LOR 1: 7.4 ± 0.2, LOR 2: 7.5 ± 0.2; WBC (10^9^/L): Veh: 5.3 ± 0.6, LOR 1: 7.1 ± 0.8, LOR 2: 5.1 ± 0.5).

**Table 3:** Summary of 28-day lorcaserin treatment on clinical chemistry and major organ weights

	Clinical chemistry								Percent organ weights			
	ALB	GLOB	AST	ALT	ALP	TBIL	BUN	CRE	Brain	Kidneys	Heart	Whole liver
Vehicle	40 ± 2	40 ± 2	117 ± 20	59 ± 13	176 ± 15	3.2 ± 0.8	5.9 ± 0.5	50.6 ± 5.7	0.30 ± 0.01	0.46 ± 0.02	0.26 ± 0.01	2.64 ± 0.09
Lorcaserin 1 mg/kg	40 ± 2	37 ± 2	106 ± 10	30 ± 3	175 ± 13	3.4 ± 0.6	5.4 ± 0.5	42.0 ± 2.4	0.29 ± 0.01	0.49 ± 0.01	0.25 ± 0.01	2.79 ± 0.12
Lorcaserin 2 mg/kg	33 ± 4	39 ± 3	90 ± 16	31 ± 7	160 ± 24	2.6 ± 0.6	5.1 ± 0.4	39.6 ± 8.7	0.30 ± 0.02	0.54 ± 0.03	0.27 ± 0.01	2.61 ± 0.09

These values all fall within normal range for SD rats (Petterino and Argentino-Storino 2006). ALB, albumin (g/L); GLOB, globulin (g/L); AST, aspartyl transaminase (U/L); ALT, alanine transaminase (U/L); ALP, alkaline phosphatase (U/L); TBIL, total bilirubin (*μ*mol/L); BUN, blood urea nitrogen (mmol/L); CRE, creatinine (*μ*mol/L).

**Figure 3:**
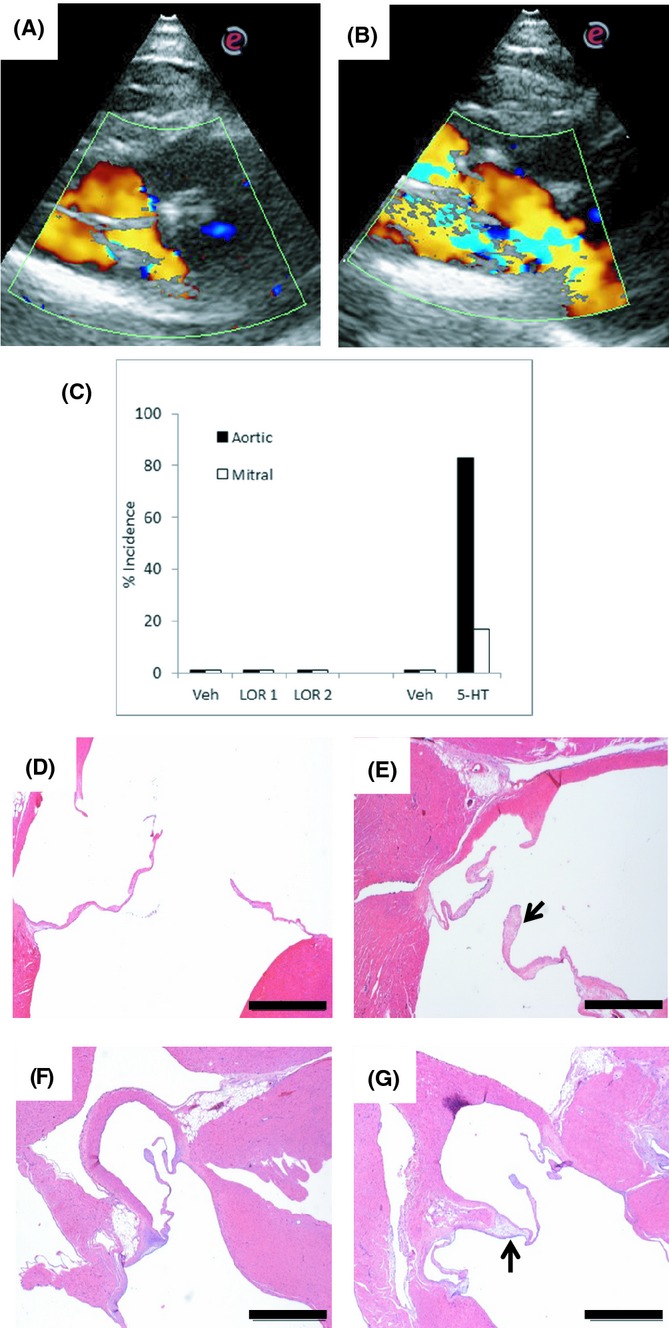
Representative echocardiographic images showing normal flow through the aortic valve with normal antegrade flow represented in yellow (A) and turbulent regurgitant flow in blue in an abnormal valve (B). Image (A) was taken from a vehicle-treated rat, (B) from a 28 day 5-HT-treated rat. (C) Graph to summarize the percent incidence of regurgitant flow across the aortic and mitral valves from all study animals. (D) Normal aortic valve, taken from a lorcaserin vehicle control rat fed high-fat diet. (E) Aortic valve, taken from a lorcaserin vehicle control rat fed high-fat diet. Note the mild multifocal thickening of valve spongiosa by moderately cellular myxomatous and collagenous matrix. (F) Normal aortic valve, taken from a vehicle control rat fed regular diet. (G) Aortic valve, taken from a 28 day 5-HT-treated rat fed regular diet. The arrow denotes expansion of spongiosa at base of valve cusp by myxomatous stroma. Scale bar = 1 mm.

In a further echocardiography study, the effect of treatment on cardiac function and regurgitation of the aortic and mitral valves was qualitatively assessed by color and pulsed wave Doppler flow (see Fig.3A and B). There was no evidence in any treatment group of aortic and mitral valve regurgitation (Fig.3A). In contrast, in a control cohort of rats treated for 28 days with 5-HT (60–75 mg/kg i.p. × 28 days; Elangbam et al. 2008), five of six rats were identified with evidence of aortic valve regurgitation (Fig.3B and C). Subsequent cardiac histopathology failed to identify any cardiac abnormalities induced by 28-day lorcaserin treatment that was distinct from vehicle-treated DIO rats. Some mild occurrence of lesions involving aortic, right atrioventricular (AV), and left AV valves were present in all treatment groups, including control animals. The predominant valve lesion in all affected animals was localized or generalized expansion of valvular spongiosa by myxomatous matrix, with involvement of either or both of the base or distal aspect of the valve leaflets. Contribution of collagenous or chondroid matrix to valve thickening was evident in few animals (see Fig.3D and E). Rats treated with the 28-day 5-HT regimen had similar localized or generalized thickening of aortic and right and left AV valves, predominately by myxomatous matrix. In aortic valve, lesions were consistently located at the base of cusps and were subjectively more severe in 5-HT-treated rats than in those treated with vehicle alone (see Fig.3F and G).

Major organs were collected from all rats at the completion of the 14 day washout phase. There was no effect of treatment on any organ weight expressed either in grams (data not shown) or as a percentage of total body weight (Table3). For example, liver weight expressed as a % of total body weight ranged from 2.6 ± 0.1% to 2.8 ± 0.1% across all three treatment groups.

### Characterization of lorcaserin pharmacokinetics in the DIO model

**Figure 4:**
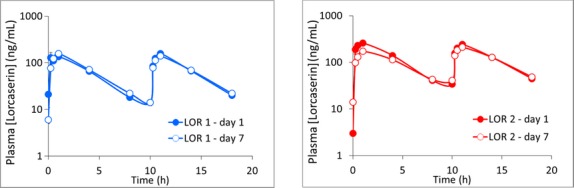
Blood levels of lorcaserin following treatment with either 1 or 2 mg/kg SC b.i.d. measured in DIO rats on DAY 1 and DAY 7 of treatment. *N* = 4 rats per dose (see Table4 for pharmacokinetics measures).

Prior to study start, there were no detectable levels of lorcaserin in the blood plasma. Acute administration of lorcaserin (1–2 mg/kg SC) resulted in a dose-related increase in drug *C*_max_ and AUC_0–8h_ that was proportional to dose (see Table4; Fig.4). *C*_min_ and *C*_max_ levels on DAY 1 were calculated to be in the range 13–160 ng/mL (1 mg/kg) and 34–264 ng/mL (2 mg/kg) and similar between am and pm dosings.

**Table 4:** Summary of pharmacokinetics parameters derived from 7-day treatment with lorcaserin (1–2 mg/kg SC × 2 daily). *N* = 4 per dose

		*C*_min_ (ng/mL)		*C*_max_ (ng/mL)		*T*_1/2_ (h)		AUC_0–8 h_ (h × ng/mL)	
		am	pm	am	pm	am	pm	am	pm
Lorcaserin 1 mg/kg SC	Day 1	–	14 ± 4	143 ± 35	158 ± 17	2.6 ± 0.2	2.3 ± 0.2	492 ± 67	582 ± 61
	Day 7	6 ± 1	14 ± 3	159 ± 15	139 ± 18	2.4 ± 0.2	2.5 ± 0.1	599 ± 50	560 ± 51
Lorcaserin 2 mg/kg SC	Day 1	–	34 ± 5	264 ± 22	244 ± 21	2.6 ± 0.1	2.6 ± 0.2	1105 ± 96	1069 ± 50
	Day 7	14 ± 4	54 ± 12	227 ± 17	212 ± 19	2.8 ± 0.2	2.9 ± 0.3	1089 ± 92	952 ± 116

After 7 days of dosing, these pharmacokinetic parameters essentially remained unchanged, for example there was no significant difference on the lorcaserin AUC_0-8 h_ measured am or pm between day 1 or day 7 (see Table4; Fig.4), indicating that over the course of the 7-day treatment period there was no evidence for drug accumulation or autoinduction. Thus drug exposure at both lorcaserin doses remained consistent over 1 week of treatment.

## Discussion

Advantages of obesity models such as the DIO rat over monogenetic models for the study of drugs to treat obesity have been identified for they better reflect the polygenic and heterogeneous causal nature of obesity (Halford et al. 2010; Vickers et al. 2011; Nilsson et al., 2012; Fellman et al. 2013). The DIO model in the present study used male Sprague–Dawley rats fed a high-fat diet (D12492, Research Diet) consisting of a fat source of soybean oil and particularly lard, which has been widely shown to produce a reliable obese phenotype (Ghibaudi et al. 2002; Buettner et al. 2006). Thus, over 12 weeks on the D12492 diet, animals developed increased body weight due to the elevated adiposity, primarily localized to the visceral fat compartment, dyslipidemia (elevated cholesterol), glucose intolerance following an oral load, and likely hepatic steatosis. Collectively, these observations indicate that this DIO procedure has good face validity as a model of human obesity (Vickers et al. 2011; Nilsson et al., 2012; Fellman et al. 2013). Both MRI and MRS are increasingly being used in clinical practice to determine fat distribution in general, and liver fat content specifically, and applying these imaging methods add further translational value to the DIO rat model (Springer et al. 2010).

The primary purpose of the present study was to evaluate the effects of the 5-HT_2C_ receptor agonist lorcaserin in the DIO model. This allowed us to establish the translatability of our results to the clinical results reported for this drug, particularly in obese patients without type 2 diabetes (Smith et al. 2008, 2010; Fidler et al. 2011). The clinical findings from these studies over a 3–12 month study period was a decline of approximately 3 kg from placebo-corrected baseline, equivalent to a 3% reduction in body mass. Thus, the current reduction of 3.0–5.2% produced by lorcaserin 1–2 mg/kg SC b.i.d. relative to vehicle is of a similar magnitude. The present studies also incorporated an estimation of the plasma drug exposure likely attained under the present dose regimen. Actual drug levels appeared consistent over 7 days of repeated treatment. Estimation of plasma *C*_min_ and *C*_max_ (1 mg/kg b.i.d. 6–158 ng/mL; 2 mg/kg b.i.d. 14–260 ng/mL) overlapped the reported plasma levels at steady state across two Phase III clinical trials at the 10 mg b.i.d. dose (∼44 ng/mL; Arena Pharmaceuticals 2010). The effect of lorcaserin treatment on the elevated plasma lipid profile was modest, which again somewhat reflects clinical findings. Thus, within-day group comparisons at the completion of treatment revealed significant decreases in cholesterol levels following both lorcaserin doses, although correction for pretreatment baseline levels attenuated the magnitude of these effects. Under the present dose regimen, lorcaserin failed to normalize the elevated blood glucose response to an oral load.

Through the use of noninvasive QMR imaging, a longitudinal assessment of body composition was also conducted in this study. While lean mass was unaffected, lorcaserin produced a dose-related decrease in fat mass showing selectivity for this tissue type, although we could not discriminate between visceral and subcutaneous fat loss using this methodology – the MRI technique was restricted to early characterization of the DIO model. This selective effect on adiposity is consistent with the report of Thomsen et al. (2008) who described a similar selective effect of lorcaserin in a 28-day study in Levin rats fed a high-fat diet. However, the effect sizes on body weight loss between the present study and the studies reported by Arena researchers (Smith et al., 2008; Thomsen et al. 2008), are somewhat different, that is, 3–5% compared to 8–10%. This difference almost certainly reflects the respective dosing regimens and distinct plasma [drug] exposures between the studies. The present study utilized a dose regimen that resulted in plasma concentrations similar to those found in the human trials, and a magnitude of weight change similar to that observed clinically. Since we have reported signs of malaise and motor impairment in rats treated with lorcaserin at doses in excess of 3 mg/kg (Higgins et al. 2012, 2013a), these additional factors may have contributed to the apparent greater weight loss reported under the higher dose regimen used in other studies (Smith et al., 2008; Thomsen et al. 2008).

Previous research has indicated that 5-HT_2C_ receptor agonists, including lorcaserin, reduce food intake through behavioral processes including motivation and satiety (Clifton et al. 2000; Somerville et al. 2007; Fletcher et al. 2010; Higgins et al. 2012, 2013a, b), although direct effects on metabolic circuitry have also been described (Heisler et al. 2002). The present studies demonstrate that lorcaserin produced a modest decrease in daily food intake, which declined in magnitude over the treatment study phase (note also Smith et al., 2008; Thomsen et al. 2008). Nonetheless, comparing treatment group differences in total food intake over the 28-day treatment period, lorcaserin produced a mean decrease in intake of 31 g (1 mg/kg b.i.d.) and 67 g (2 mg/kg b.i.d.) food relative to vehicle-treated controls, which given an energy density of 5.24 kcal/g (equivalent to 21.9 kJ/g) corresponded to 679 kJ and 1467 kJ respectively. Based on an assumption that an energy intake of 20 kJ corresponds to a gram of body weight gain in the rat (Farrell and Williams 1989; Thornton-Jones et al. 2006), this suggests that the deficit on cumulative food intake should equate to 34 g and 73 g of body weight change at the 1 and 2 mg/kg b.i.d. treatment groups. That the animals actually lost almost half this body weight value, that is, 17 g and 40 g, at least indicates that weight loss is entirely accountable by reduced energy intake, rather than increased energy utilization. As such, these data are consistent with those from an energy balance study in humans which concluded that lorcaserin reduces body mass by reducing energy intake rather than enhancing energy expenditure (Martin et al. 2011).

In a final part of this study, we investigated the impact of 28-day lorcaserin treatment on various safety parameters. Lorcaserin failed to influence cardiac function as judged by no retrograde blood flow being evident across the aortic or mitral valves measured by echocardiography. In contrast, in rats treated for 28 days with 5-HT under a regimen of 60–75 mg kg^−1^ day^−1^ (see Elangbam et al. 2008), some aortic valve regurgitation was evident in 5/6 rats. This result confirms that a 28-day regimen of drug treatment is sufficient to detect a functional cardiac abnormality, including valvulopathy. What was harder to interpret was the cardiac histopathology, where some valvular lesions were evident in the majority of both control and lorcaserin-treated rats. That this effect was found in control and lorcaserin-treated rats may suggest an impact of the high-fat diet itself. A previous study has demonstrated cardiac inclusions in rats fed a high-fat diet for 15 weeks (Leopoldo et al. 2010). By the time tissues were taken from subjects in the present study, the animals had been maintained on the experimental diet for an even longer period of almost 20 weeks. Thus, the interpretation of data from this study is that structural cardiac abnormalities relate to diet rather than drug treatment per se. There were no treatment effects on markers of liver function, blood cell counts or major organ weights.

While several reports have documented the rat DIO as a model for human obesity, relatively few studies have focused on the predictive value of such models through backtranslating clinical findings of pharmacological treatments to the preclinical model. Given the continuing clinical failure of NCE's due to the inadequate efficacy prediction (Kola and Landis 2004; Enna and Williams 2009; Hay et al. 2014), such studies have obvious value. Vickers et al. (2011) have recently highlighted the predictive validity of the DIO model, by showing that the magnitude of body weight change produced by anorectic drugs of multiple pharmacological classes in a 28-day DIO model appears to correspond well to clinical outcomes. The present study adds to this dataset. Taken together, these DIO studies reflect the modest clinical change produced by lorcaserin, which is a small improvement over the effect of orlistat, but is less than that induced by topiramate/phentermine (Qnexa®), and probably the CB1 antagonist rimonibant and the mixed NA/5-HT reuptake inhibitor sibutramine (Kennett and Clifton 2010; Heal et al. 2013). The effect size of lorcaserin on body weight loss in the present DIO rat study was smaller than that reported by others (Smith et al., 2008; Thomsen et al. 2008) although in these latter studies a contribution of nausea/malaise to the observed weight change seems likely. Indeed nausea and malaise are primary side effects reported for lorcaserin in the clinic, especially at supratherapeutic doses (Chan et al., 2013; Schram et al. 2011). Thus, assuming due consideration is paid to clinically unacceptable contributors, such as malaise and sedation, to the reduction in body weight gain, the DIO model may have predictive value to determine clinical outcomes of an NCE.

It is interesting to speculate whether the 3.2% weight change produced by lorcaserin represents the optimal effect size of this drug, or whether alternative 5-HT_2C_ receptor agonists may improve on this measure perhaps by virtue of greater functional selectivity at the 5-HT_2C_ receptor (Urban et al. 2007), and/or better tolerability profile through minimizing adverse effects such as nausea/malaise (Higgins et al. 2013a, b). Given the apparent predictive value of the DIO model, this should be testable assuming due consideration is paid to side effects related either to tolerability or safety. Of final note, while the current studies were not designed to determine the behavioral mechanisms underlying the anorectic effect of lorcaserin, an understanding of the antiobesity mechanisms of lorcaserin would be advantageous and perhaps help to identify the obese patient populations most sensitive to the anorectic effects of 5-HT_2C_ receptor agonists including lorcaserin.

## Abbreviations

5-HT: 5-hydroxytryptamine (serotonin)
DIO: diet-induced obesity
QMR: quantitative magnetic resonance
FDA: Food and Drug Administration
EMA: European medicines agency
LDL-C: low-density lipoprotein-cholesterol
SC: subcutaneous
AV: atrioventricular
AUC: area under curve
RBC: red blood cell
WBC: white blood cell
*C*_min_: minimum drug plasma concentration
*C*_max_: maximum drug plasma concentration
NA: noradrenaline
NCE: new chemical entity
OGTT: oral glucose tolerance test
